# Ocular adverse events associated with antibody-drug conjugates for cancer: evidence and management strategies

**DOI:** 10.1093/oncolo/oyae177

**Published:** 2024-07-24

**Authors:** Grace K Dy, Asim V Farooq, Joann J Kang

**Affiliations:** Department of Medicine, Roswell Park Comprehensive Cancer Center, Buffalo, NY 14203, United States; Department of Ophthalmology and Visual Science, University of Chicago Medical Center, Chicago, IL 60612, United States; Department of Ophthalmology and Visual Sciences, Montefiore Medical Center, Albert Einstein College of Medicine, Bronx, NY 10461, United States

**Keywords:** antibody-drug conjugate, cancer, ocular adverse events

## Abstract

Antibody-drug conjugates (ADCs) are a fast-growing class of cancer drugs designed to selectively deliver cytotoxic payloads through antibody binding to cancer cells with high expression of the target antigen, thus reducing systemic exposure and minimizing off-target effects. However, ADCs are associated with various ocular adverse events (AEs) that may impact treatment administration and patient outcomes. In this review, we provide a summary of ocular AEs associated with approved and investigational ADCs, recommendations for the mitigation and management of ocular AEs, current guidelines and expert opinions, and recommendations for clinical practice. A literature search was performed, using PubMed and Google Scholar, for English-language articles published between January 1985 and January 2023 to identify studies reporting ocular AEs associated with ADC use. Search terms included generic and investigational names of all identified ADCs, and further searches were performed to identify strategies for managing ADC-associated ocular AEs. ADC-associated ocular AEs include symptoms such as blurred vision and foreign-body sensation and signs such as corneal fluorescein staining, corneal pseudomicrocysts, and conjunctivitis. Reported management strategies include ADC dose modification (eg, dose delay or reduction), cool compresses, artificial tears, topical vasoconstrictors, and topical steroids. Although ADC dose modification appears to be beneficial, the preventive and/or therapeutic benefits of the remaining interventions are unclear. Although the exact mechanisms are not fully understood, most ADC-associated ocular AEs are reversible with dose delay or dose reduction. Management of ocular AEs requires a multidisciplinary approach to minimize treatment discontinuation and optimize clinical outcomes.

## Teaser

Antibody-drug conjugates are associated with several ocular adverse events, all of which can interrupt treatment and affect clinical outcomes. This review provides a summary of the ocular adverse events associated with approved and investigational ADCs and provides recommendations for their management.

**Implications for practice**

Antibody-drug conjugates (ADCs) are a rapidly emerging class of therapeutic agents designed to selectively reach cancer cells with limited damage to noncancerous cells. Nevertheless, ADCs are associated with several ocular adverse events (AEs), all of which can interrupt treatment and impact clinical outcomes. Although the exact mechanism of ADC-associated ocular AEs is still unclear, ADC uptake into normal corneal epithelial cells via nonspecific endocytosis may play a role. This review provides a summary of the ocular AEs associated with approved and investigational ADCs and provides recommendations for their management.

## Introduction

**Figure 1.**
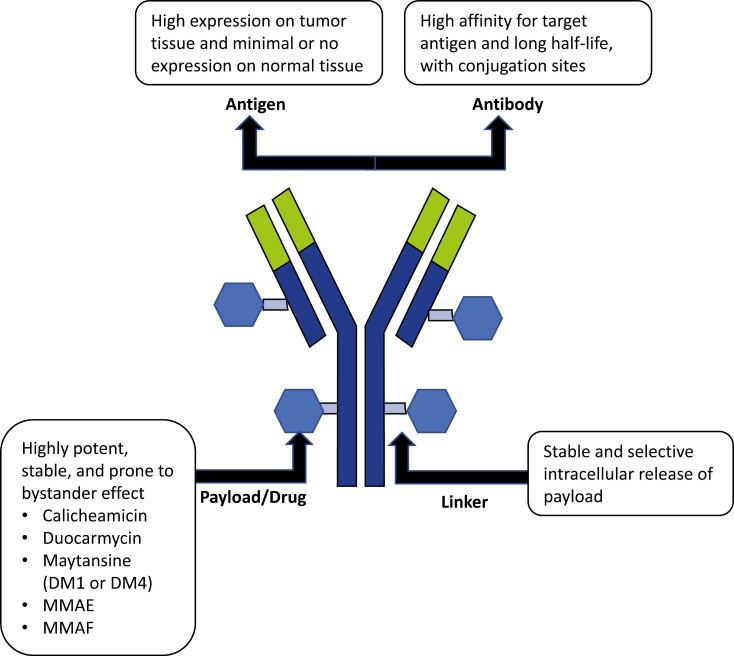
Structure of an antibody-drug conjugate. MMAE, monomethyl auristatin E; MMAF, monomethyl auristatin-F.

Antibody-drug conjugates (ADCs) are anticancer agents designed to selectively deliver a payload (also known as a “warhead”) directly to cancer cells,^1,2^ thereby improving the efficacy of chemotherapy while reducing systemic exposure and subsequent off-target effects.^1,3^ ADCs consist of 3 main components: (1) a monoclonal antibody (mAb) with specificity for a tumor cell surface protein; (2) a payload; and (3) a linker that connects the 2 substances, which must be stable in circulation (Figure 1).^1,2,4,5^ Payloads are categorized based on their mode of action; these include agents that disrupt tubulin organization (eg, maytansinoid DM4, auristatin metabolite monomethyl auristatin-F [MMAF], monomethyl auristatin E [MMAE] or mertansine [DM1], AS269), affect deoxyribonucleic acid (DNA) replication (eg, topoisomerase I inhibitor, duocarmycin), or inhibit transcription (eg, amatoxins).^6^ Specific and efficient binding of the mAb to its target antigen on tumor cells, while avoiding normal cells, is essential to minimize off-target toxicity.^3^ After the mAb binds to its target antigen, the receptor-ADC complex is internalized via endocytosis, and the linker is biochemically cleaved or spontaneously degrades to free the payload in sufficient concentrations to act on its intracellular or extracellular target.^7-9^ Cleavage of the payload may also occur in an off-target fashion due to the presence of extracellular enzymes, hence the important role of linker technology in maintaining the stability and efficiency of payload delivery.^9^

The increasing number of approved ADCs means these agents are becoming important therapeutic options for oncologists to consider when treating patients with cancer^10^; however, their diverse spectrum of adverse events (AEs) should also be considered, as these may cause a delay in or discontinuation of treatment, and in turn, may impact patient outcomes. Therefore, successful AE management in patients receiving ADC treatment is essential to prevent such treatment interruption or discontinuation.

Ocular AEs are particularly prevalent during ADC treatment, although they may also develop with other targeted anticancer therapies.^10,11^ The most commonly observed ocular AEs with ADCs are corneal or surface AEs and may include symptoms of blurred vision and/or foreign-body sensation and clinical signs such as corneal fluorescein staining, pseudomicrocysts (also known as microcyst-like epithelial changes), and conjunctivitis; however, intraocular AEs and other sporadic events may also occur and are described herein.^12^

This review details ocular AEs associated with approved and investigational ADCs and provides oncologists with recommendations for the mitigation and management of ADC-associated ocular AEs based on current guidelines and expert opinions. Recommendations for clinical practice and future research are also included.

## Methods

A search of the literature was conducted, using PubMed and Google Scholar, for English-language articles published between January 1985 and January 2023, to identify ocular AEs that occurred during anticancer treatment with an ADC. The search terms included the generic and investigational names of all identified ADCs and were designed to identify clinical trial results. Further searches were conducted to identify management strategies used to mitigate and manage ocular AEs during ADC therapy. Hand searches of literature review articles and a search of clinicaltrials.gov were also conducted to ensure as many ADCs were included as possible.

## Mechanisms of ADC-associated ocular AEs: on-target and off-target toxicity

Although the exact mechanism(s) by which ADCs cause ocular AEs is not known, uptake of ADCs and their payloads into normal, noncancerous cells may be attributed to on-target or off-target toxicity. On-target toxicity may occur when the target antigen (eg, HER2) is expressed on normal corneal epithelial cells and contributes to target-dependent uptake of ADCs (eg, trastuzumab emtansine and trastuzumab duocarmazine).^13^ Conversely, off-target toxicity may arise due to the nonspecific uptake of ADCs and their payloads (eg, belantamab mafodotin). ADCs or their cytotoxic payloads may enter normal, noncancerous cells in several ways, including receptor-mediated endocytosis via Fcγ receptors, target antigens, or C-type lectin receptors (CLRs); nonspecific endocytosis via macro/micropinocytosis; or passive or facilitated diffusion of the payload into the cell as the result of deconjugation from the antibody and/or a compromised neighboring cell (ie, bystander effect).^9^ Mounting evidence suggests that ADC-associated ocular AEs are predominantly target-independent (ie, off-target) and attributed to the payload rather than the antigen expressed on normal cells.^9^ Further studies to inhibit or modulate off-target toxicity (eg, macropinocytosis-mediated internalization of the ADC) may help mitigate ocular AEs in the future. Preclinical models have demonstrated that altering the hydrophobicity of the ADC, such as by pegylation, or conferring neutral/negatively charged residues, may influence such off-target toxicity.^14^

## Ocular AEs with ADCs in clinical use and in development

Several ADCs have been approved or are in clinical development for the treatment of patients with various cancer types, including lymphoma, multiple myeloma (MM), leukemia, urothelial carcinoma (UC), ovarian, fallopian tube, peritoneal, cervical, breast, and gastric cancer, and non-small cell lung cancer (NSCLC).

This section provides an overview of ADCs that are associated with ocular AEs during cancer treatment. These AEs are often identified in studies by standard ophthalmologic tests, including a slit-lamp examination and best-corrected visual acuity (BCVA) test. Slit-lamp examination uses a low-power microscope combined with a high-intensity light to examine the eyes, including the eyelids, cornea, conjunctiva, sclera, iris, lens, and retina. BCVA refers to the smallest letters one can read on a standardized chart (such as a Snellen chart) with the best possible glasses or contact lens prescription. Description of common ocular AEs following ADC use and definitions of their severity (grades 1-5, per Common Terminology Criteria for Adverse Events [CTCAE] version 5.0) are included in Table 1. Furthermore, a comprehensive listing of ocular AE rates with approved and investigational ADCs are in given Tables 2 and 3, respectively. Following is a discussion of approved and investigational ADCs, organized by payloads, with a focus on therapies that resulted in grade ≥3 ocular AEs and/or in treatment discontinuation during the study.

**Table 1.** Common ocular AEs associated with use of ADCs by grade.^15^

CTCAE term	Definition	Grade 1	Grade 2	Grade 3	Grade 4	Grade 5
Keratitis	A disorder characterized by inflammation of the cornea of the eye	Asymptomatic; clinical or diagnostic observations only; intervention not indicated	Symptomatic; moderate decrease in visual acuity (best corrected visual acuity 20/40 and better or 3 lines or less decreased vision from known baseline)	Symptomatic with marked decrease in visual acuity (best corrected visual acuity worse than 20/40 or more than 3 lines of decreased vision from known baseline, up to 20/200); corneal ulcer; limiting self-care ADL	Perforation; best corrected visual acuity of 20/200 or worse in the affected eye	—
Blurred vision	A disorder characterized by visual perception of unclear or fuzzy images	Intervention not indicated	Symptomatic; moderate decrease in visual acuity (best corrected visual acuity 20/40 and better or 3 lines or less decreased vision from known baseline); limiting instrumental ADL	Symptomatic with marked decrease in visual acuity (best corrected visual acuity worse than 20/40 or more than 3 lines of decreased vision from known baseline, up to 20/200); limiting self-care ADL	Best corrected visual acuity of 20/200 or worse in the affected eye	—
Cataract	A disorder characterized by partial or complete opacity of the crystalline lens of one or both eyes. This results in a decrease in visual acuity and eventual blindness if untreated	Asymptomatic; clinical or diagnostic observations only; intervention not indicated	Symptomatic; moderate decrease in visual acuity (best corrected visual acuity 20/40 and better or 3 lines or less decreased vision from known baseline); glare symptoms affecting instrumental ADL	Symptomatic with marked decrease in visual acuity (best corrected visual acuity worse than 20/40 or more than 3 lines of decreased vision from known baseline, up to 20/200); limiting self-care ADL	Best corrected visual acuity of 20/200 or worse in the affected eye	—
Conjunctivitis	A disorder characterized by inflammation, swelling, and redness of the conjunctiva of the eye	Asymptomatic or mild symptoms; intervention not indicated	Symptomatic; moderate decrease in visual acuity (best corrected visual acuity 20/40 and better or 3 lines or less decreased vision from known baseline)	Symptomatic with marked decrease in visual acuity (best corrected visual acuity worse than 20/40 or more than 3 lines of decreased vision from known baseline, up to 20/200); limiting self-care ADL	Best corrected visual acuity of 20/200 or worse in the affected eye	—
Dry eye	Disorder characterized by dryness of the cornea and conjunctiva	Asymptomatic; clinical or diagnostic observations only; symptoms relieved by lubricants	Symptomatic; moderate decrease in visual acuity (best corrected visual acuity 20/40 and better or 3 lines or less decreased vision from known baseline)	Symptomatic with marked decrease in visual acuity (best corrected visual acuity worse than 20/40 or more than 3 lines of decreased vision from known baseline, up to 20/200); limiting self-care ADL	—	—
Eye pain	A disorder characterized by a sensation of marked discomfort in the eye	Mild pain	Moderate pain; limiting instrumental ADL	Severe pain; limiting self-care ADL	—	—
Night blindness	A disorder characterized by an inability to see clearly in dim light	Symptomatic but not limiting ADL	Symptomatic; moderate decrease in visual acuity (best corrected visual acuity 20/40 and better or 3 lines or less decreased vision from known baseline); limiting instrumental ADL	Symptomatic with marked decrease in visual acuity (best corrected visual acuity worse than 20/40 or more than 3 lines of decreased vision from known baseline, up to 20/200); limiting self-care ADL	Best corrected visual acuity of 20/200 or worse in the affected eye	—
Photophobia	A disorder characterized by fear and avoidance of light	Symptomatic but not limiting ADL	Limiting instrumental ADL	Limiting self-care ADL	——	—

Abbreviations: ADC, antibody-drug conjugate; ADL, activities of daily living; AE, adverse event; CTCAE, Common Terminology Criteria for Adverse Events.

**Table 2.** Summary of ocular AEs and tumor response rates reported in clinical studies of ADCs in current clinical use.

Study	Prophylactic strategies to mitigate ocular AEs	ADC dosage (no. of patients)	Ocular AEs	Median time to onset	Response rates
*Belantamab mafodotin—RRMM* BCMA-targeting IgG1 mAb conjugated to the microtubule inhibitor MMAF via a protease-resistant maleimidocaproyl linker					
DREAMM-1^16^	Steroid eye drops (prednisolone phosphate 1% or dexamethasone 0.1% QID for 4 days, starting 1 day predose)	3.4 mg/kg Q3W (*n* = 35)	Blurred vision: 46% (grades 1-2) Dry eye: 31% (grades 1-2); 3% (grade 3) Photophobia: 23% (grades 1-2) Increased lacrimation: 11% (grades 1-2) Keratitis: 3% (grades 1-2); 6% (grade 3) Eye pain: 3% (grades 1-2); 3% (grade 3) Keratopathy: 6% (grades 1-2) Eye pruritus: 3% (grades 1-2) Night blindness: 3% (grades 1-2)	Corneal AEs: 23 (range: 1-84) days	ORR: 60% (95% CI 42.1-76.1) CR: *n* = 2/35 (6%) Very good PR: *n* = 15/35 (43%) PR: *n* = 3/35 (9%)
DREAMM-2^17,18^	Steroid eye drops (prednisolone acetate 1%, prednisolone sodium phosphate 1%, dexamethasone 0.1%, or equivalent, one drop QID for 7 days, starting 1 day predose) and preservative-free lubricant eye drops (one drop, 4-8 times daily, starting on cycle 1, day 1 until end of treatment) in both eyes. Use of contact lenses was prohibited during the study, and cooling eye masks could be applied at the start of infusion.	2.5 mg/kg Q3W (*n* = 97)	Keratopathy^a^: 43% (grades 1-2); 27% (grade 3) Blurred vision^b^: 18% (grades 1-2); 4% (grade 3) Dry eye^c^: 13% (grades 1-2); 1% (grade 3)	First occurrence of pseudomicrocysts: 37 (range:19-143) days Blurred vision: 51.5 (range: 6-339) days Dry eye: 42 (range: 12-51) days BCVA 20/50 or worse: 66 (range: 20-442) days	ORR: 31% (97.5% CI: 20.8-42.6) Very good PR: *n* = 18/97 (19%)
		3.4 mg/kg Q3W (*n* = 99)	Keratopathy^a^: 54% (grades 1-2); 21% (grade ≥3) Blurred vision^b^: 28% (grades 1-2); 2% (grade 3) Dry eye^c^: 23% (grades 1-2)	NR	ORR: 34% (97.5% CI: 23.9-46.0) Very good PR: *n* = 20/99 (20%)
*Brentuximab—relapsed or refractory Hodgkin lymphoma*					
Case report^19^	NR	NR	Panuveitis	15 days	—
Case report^20^	NR	1.8 mg/kg Q3W	Purtscher-like retinopathy	Nearly 3 weeks	—
Case report^21^	NR	NR	Uveitis	3 weeks	—
*Enfortumab vedotin—locally advanced or metastatic urothelial carcinoma* Nectin-4-targeting IgG1 mAb conjugated to the microtubule inhibitor MMAE via a protease cleavable linker					
EV-201^22^	NR	1.25 mg/kg on days 1, 8, and 15 of 28-day cycle (*n* = 125)	Dry eye: 19% (grades 1-2) Increased lacrimation: 14% (grades 1-2) Blurred vision: 12% (grades 1-2)	NR	ORR: 44% (95% CI: 35.1-53.2) CR: *n* = 15/125 (12%) PR: *n* = 40/125 (32%)
EV-301^23^	None	1.25 mg/kg on days 1, 8, and 15 of 28-day cycle (*n* = 296)	Dry eye: 15.9% (any grade); 0.7% (grade 3) Blurred vision: 4.1% (grades 1-2) Corneal disorders: 0.7% (grades 1-2)	Dry eye: 1.9 (range: 0.3-9.7) months Blurred vision: 2.4 (range: 0.1-5.1) months Corneal disorders: 4.3 (range: 1.9-6.8) months	ORR: 40.6% (95% CI:34.9-46.5) CR: *n* = 14/288 (4.9%) PR: *n* = 103/288 (35.8%)
*Mirvetuximab soravtansine—FRα-positive ovarian epithelial, fallopian tube, or primary peritoneal cancer* FRα-targeting IgG1 subtype 2 mAb conjugated to the microtubule inhibitor DM4 via a cleavable linker					
FORWARD I^24^	Steroid eye drops (1% prednisolone, 6 times daily on days 1-4 and QID on days 5-8 of each cycle) and preservative-free, lubricating artificial tears (daily, as directed by the product label or treating physician)	6 mg/kg Q3W (*n* = 243)	Blurred vision: 42.0% (any grade); 2.5% (grade ≥3) Keratopathy: 32.5% (any grade); 1.2% (grade ≥3) Dry eye: 25.9% (any grade); 1.2% (grade ≥3) Decreased visual acuity: 19.3% (grades 1-2)	NR	ITT population ORR: 22% High FRα subset ORR: 24%
SORAYA^25^	Preservative-free lubricating artificial tears (daily) and steroid eye drops (starting 1 day predose and continuing through day 8 of each cycle)	6 mg/kg Q3W (*n* = 106)	Blurred vision: 41% (any grade); 6% (grade ≥3) Keratopathy: 29% (any grade); 9% (grade ≥3) Photophobia: 13% (grades 1-2)	Blurred vision: 1.3 (range: 0.0-9.9) months Keratopathy: 1.5 (range: 1.1-8.6) months	Investigator-assessed ORR: 32.4% (95% CI: 23.6-42.2) BICR-assessed ORR: 30.2% (95% CI: 21.3-40.4) Investigator-assessed CR: *n* = 5/105 (4.8%) PR: *n* = 29/105 (27.6%) BICR-assessed CR: *n* = 6/96 (6.3%) PR: *n* = 23/96 (24.0%)
MIRASOL^26^	NR	6 mg/kg Q3W (*n* = 227)	Ocular AEs: 56% (low grade); 14% (grade ≥3)	NR	Investigator-assessed ORR: 42.3% (95% CI: 35.8-49.0) CR: 12/227 (5.3%) PR: 84/227 (37.0%) BICR-assessed ORR: 36.1% (29.9-42.7)
*Tisotumab vedotin—recurrent or metastatic cervical cancer* Tissue factor-targeting IgG1-kappa mAb conjugated to the microtubule inhibitor MMAE via a protease cleavable linker					
innovaTV 201^27^	Preservative-free lubricating eye drops (from the start until the end of treatment), local ocular vasoconstrictor eye drops (immediately prior to the start of infusion), cooling eye pads (during infusion), and steroid eye drops (for 3 days beginning on the day of infusion). Use of contact lenses was avoided and stricter dose modification guidance for ocular events was provided.	2.0 mg/kg Q3W (*n* = 55)	Conjunctivitis: 42% (any grade); 2% (grade ≥3) Dry eye: 24% (grades 1-2) Ulcerative keratitis: 7% (grades 1-2) Blepharitis: 5% (grades 1-2) Keratitis: 5% (grades 1-2)	NR	Investigator-assessed ORR: 24% (95% CI: 13-37) IRC-assessed ORR: 22% (95% CI: 12-35) Investigator-assessed CR: *n* = 0/55 (0%) PR: *n* = 13/55 (24%) IRC-assessed CR: *n* = 1/55 (2%) PR: *n* = 11/55 (20%)
innovaTV 206^28^	Preservative-free lubricating eye drops (throughout the study), steroid eye drops (before the start of the infusion and for the first 3 days of each treatment cycle), a local ocular vasoconstrictor (before infusion), and eye cooling pads (during infusion)	2.0 mg/kg Q3W (*n* = 17)	Conjunctivitis: 17.6% (grades 1-2) Allergic conjunctivitis: 5.9% (grades 1-2) Scleritis: 5.9% (grades 1-2) Hordeolum: 5.9% (grades 1-2) Blurred vision: 5.9% (grades 1-2)	NR	ORR: 29.4% (95% CI: 10.3-56.0) CR: *n* = 5/17 (29.4%) PR: *n* = 7/17 (41.2%)
innovaTV 204/ GOG-3023/ ENGOT-cx6^29^	Steroid eye drops (before each infusion and continued for 72 hours thereafter), local ocular vasoconstrictor eye drops (before infusion), eye cooling pads (during infusion), preservative-free lubricating eye drops (from the first dose until 30 days after the last dose), and no contact lens use (during the entire course of therapy)	2.0 mg/kg Q3W (*n* = 101)	Conjunctivitis: 26% (grades 1-2) Dry eye: 23% (grades 1-2) Keratitis: 11% (grades 1-2) Ulcerative keratitis: 2% (grade 3)	Ocular AE: 1.4 (IQR: 0.7-2.0) months	ORR: 24% (95% CI: 16-33) CR: *n* = 7/101 (7%) PR: *n* = 17/101 (17%)
*Trastuzumab deruxtecan—HER2-positive breast cancer* HER2-targeting IgG1 mAb conjugated to a topoisomerase inhibitor via a cleavable linker					
DESTINY-Breast01^30^	NR	5.4 mg/kg Q3W (*n* = 184)	Dry eye: 11.4% (any grade); 0.5% (grade 4)	NR	ORR: 61% (95% CI: 53-68) CR: 6.0% (out of 184 patients) PR: 54.9% (out of 184 patients)
DESTINY-Breast03^31,32^	NR	5.4 mg/kg Q3W (*n* = 261)	Blurred vision: 3.5% (any grade)	NR	ORR: 79.7% (95% CI: 74.3-84.4) CR: *n* = 42/261 (16.1%) PR: *n* = 166/261 (63.6%)
DESTINY-Breast04^31,33^	NR	5.4 mg/kg Q3W (*n* = 373)	Blurred vision: 4.9% (any grade)	NR	Overall population ORR: 52.3% (95% CI: 47.1-57.4) Hormone-receptor positive cohort ORR: 52.6% (95% CI: 47.0-58.0) Overall population CR: *n* = 13/373 (3.5%) PR: *n* = 183/373 (49.1%) Hormone-receptor positive cohort CR: *n* = 12/331 (3.6%) PR: *n* = 164/331 (49.5%)
*Trastuzumab emtansine—HER2-positive breast cancer* HER2-targeting IgG1 mAb conjugated to the microtubule inhibitor DM1 via a stable thioether linker					
EMILIA^34,35^	NR	3.6 mg/kg Q3W (*n* = 495)	Blurred vision: 4.5% (any grade) Conjunctivitis: 3.9% (any grade) Dry eye: 3.9% (any grade) Lacrimation increased: 3.3% (any grade)	NR	ORR: 43.6% (95% CI: 38.6%-48.6%) CR: *n* = 4/397 (1.0%) PR: *n* = 169/397 (42.6%)
KATHERINE^34,36^	NR	3.6 mg/kg Q3W (*n* = 743)	Lacrimation increased: 6.0% (any grade) Dry eye: 4.5% (any grade) Blurred vision: 3.9% (any grade) Conjunctivitis: 3.5% (any grade)	NR	NR

^a^Defined as corneal epithelium changes (± symptoms) on ophthalmic examination.

^b^Included blurred vision, diplopia, reduced visual acuity, and visual impairment.

^c^Included dry eye, ocular discomfort, eye pruritus, and foreign-body sensation in eye.

Abbreviations: ADC, antibody-drug conjugate; AE, adverse event; BCMA, B-cell maturation antigen; BCVA, best-corrected visual acuity; BICR, blinded independent central review; CI, confidence interval; CR, complete response; FRα, folate receptor alpha; HER2, human epidermal growth factor receptor-2; IgG1, immunoglobulin G1; IQR, interquartile range; IRC, independent review committee; ITT, intention-to-treat; mAb, monoclonal antibody; MMAE, monomethyl auristatin E; MMAF, monomethyl auristatin-F; NR, not reported; ORR, objective response rate; PR, partial response; Q3W, every 3 weeks; QID, 4 times daily; RRMM, relapsed or refractory multiple myeloma.

**Table 3.** Summary of ocular AEs and response rates with ADCs in recent clinical development.

Indication (no. of patients)	ADC dosage	Ocular AEs	Response rates
*Anetumab ravtansine* Mesothelin-targeting IgG1 mAb conjugated to the microtubule inhibitor DM4 via a reducible disulfide linker			
Mesothelin-positive malignant pleural mesothelioma (*n* = 163)^37^	6.5 mg/kg Q3W	Corneal disorder: 37% (grades 1-2); 2% (grade 3) Dry eye: 13% (grades 1-2)	ORR: 9.6% (95% CI: 5.6-15.2) CR: *n* = 0/166 (0%) PR: *n* = 16/166 (9.6%)
Platinum-resistant ovarian cancer (*n* = 65)^38^	5.5 or 6.5 mg/kg Q3W + pegylated liposomal doxorubicin	At least one corneal epitheliopathy event: 47.7% (grades 1-2) Corneal disorder: 29.2% (grades 1-2) Dry eye: 16.9% (grades 1-2) Blurred vision: 10.8% (grades 1-2); 3.1% (grade 3)	ORR: 27.7% (95% CI: 17.3-40.2) CR: *n* = 1/65 (1.5%) PR: *n* = 17/65 (26.2%)
Mesothelin-positive advanced pancreatic adenocarcinoma (*n* = 33)^39^	5.5 or 6.5 mg/kg Q3W + nivolumab, nivolumab-ipilimumab, or nivolumab-gemcitabine	Blurred vision: 15% (grades 1-2) Xerophthalmia: 3% (grade 1) Keratitis: 3% (grade 2)	ORR: 0% CR: *n* = 0/25 (0%) PR: *n* = 0/25 (0%)
*ARX788* HER2-targeting antibody conjugated to an anti-tubulin payload AS269 via a non-natural amino acid (para-acetyl phenylalanine)			
HER2-positive metastatic breast cancer (*n* = 69)^40^	1.5 mg/kg Q3W or 0.88-1.3 mg/kg Q4W	Corneal epitheliopathy: 46.4% (any grade); 4.3% (grade ≥3) Blurred vision: 21.7% (any grade); 2.9% (grade ≥3) Xerophthalmia: 21.7% (any grade)	ORR: 65.5% (95% CI: 45.7-82.1) PR: *n* = 33/69 (47.8%)
*Coltuximab ravtansine* CD19-targeting mAb conjugated to the microtubule inhibitor DM4 via a cleavable disulfide linker			
DLBCL (*n* = 52)^41^	55 mg/m^2^ QW for 4 weeks, then Q2W for 8 weeks + rituximab	Eye disorders: 19% (any grade) Extracorneal events: 12% (any grade) Lacrimal events: 8% (any grade) Corneal events: 6% (any grade)	ORR^a^: 31.1% (80% CI: 22.0-41.6) CR: *n* = 4/45 (8.9%) PR: *n* = 10/45 (22.2%)
DLBCL (*n* = 61)^42^	55 mg/m^2^ QW for 4 weeks, 1 week rest, then Q2W	Eye disorders: 25.0% (grades 1-2) Extracorneal eye disorders: 21.3% Dry eye: 3.3% Keratitis: 1.6% (grade 2)	ORR: 43.9% (90% CI: 90.6-57.9) CR: *n* = 6/41 (14.6%) PR: *n* = 12/41 (29.3%)
ALL (*n* = 36)^43^	55, 70, or 90 mg/m^2^ Q2W	Ocular AEs: 25% (grades 1-2) Extracorneal events: 17% Blurred vision: 14% Lacrimal disorder: 6% Keratitis: 3%	ORR: 25.5% (80% CI: 14.2-39.6) CR 55 mg/m^2^: *n* = 2/7 (29%) 70 mg/m^2^: *n* = 1/17 (6%) 90 mg/m^2^: *n* = 0/7 (0%) CR without recovery of counts 55 mg/m^2^: *n* = 0/7 (0%) 70 mg/m^2^: *n* = 2/17 (12%) 90 mg/m^2^: *n* = 0/7 (0%) PR 55 mg/m^2^: *n* = 1/7 (14%) 70 mg/m^2^: *n* = 1/17 (6%) 90 mg/m^2^: *n* = 1/7 (14%)
*Datopotamab deruxtecan (Dato-DXd)* TROP2-targeting IgG1 mAb conjugated to topoisomerase inhibitor via a tetrapeptide-based cleavable linker			
TNBC (*n* = 43)^44^	6 mg/kg Q3W	Dry eye, retinal exudates, blurred vision requiring dose reduction (incidences not reported)	ORR: 39% CR: *n* = 12/38 (31.6%) PR: *n* = 15/38 (39.5%)
NSCLC (*n* = 136)^45^	4 mg/kg or 6 mg/kg	Ocular surface toxicity: (Dato-DXd + pembrolizumab) 16% (any grade), 2% (grade ≥3); (Data-DXd + pembrolizumab + chemotherapy) 24% (any grade), 3% (grade ≥3) Majority of events were dry eye or lacrimation increase; grade ≥3 events were keratitis and dry eye.	ORR any line therapy (Dato-DXd + pembrolizumab): 38% (95% CI: 26-51) ORR any-line therapy (Dato-DXd + pembrolizumab + chemotherapy): 49% (95% CI: 37-61)
NSCLC (*n* = 299)^46^	6 mg/kg Q3W	Ocular AEs: 19% (any grade); 2% (grade ≥3) Dry eye: 6.1% (primarily grade ≤2) Increased lacrimation: 5.4%	ORR: 26.4%
*Praluzatamab ravtansine* Probody drug conjugate composed of a CD166-targeting mAb conjugated to the microtubule inhibitor DM4 via a disulfide cleavable linker			
Advanced solid tumors (*n* = 99)^47^	0.25-10 mg/kg Q3W or 4-6 mg/kg Q2W	Ocular TRAEs: 43% (any grade); 11% (grade ≥3) Keratitis: 21% (any grade); 9% (grade ≥3) Blurred vision: 16% (any grade) Dry eye: 8% (any grade) Keratopathy: 3% (any grade) Photophobia: 3% (any grade) Corneal infiltrates: 1% (any grade) Orbital cyst: 1% (any grade)	ORR: 3% (95% CI: 0.3-8.7) CR: *n* = 2/80 (3%) Unconfirmed PR: *n* = 6/80 (8%)
Advanced hormone receptor-positive/HER2-negative breast cancer (*n* = 60)^48^	7 mg/kg Q3W	Blurred vision: 42% (any grade) Ocular TRAEs: 15% (grade ≥3)	ORR: 15% for hormone receptor-positive/HER2-negative breast cancer
Advanced TNBC (*n* = 55)^48^	6 mg/kg Q3W	Ocular TRAEs: 3% (grade ≥3)	ORR: <10% for TNBC
*Trastuzumab duocarmazine* HER2-targeting mAb covalently bound via a cleavable linker to a duocarmycin prodrug			
Metastatic solid tumors (*n* = 146)^49^	1.2 mg/kg Q3W	Dry eye: 30% (grades 1-2); 1% (grade 3) Conjunctivitis: 28% (grades 1-2); 3% (grade 3) Increased lacrimation: 20% (grades 1-2) Keratitis: 17% (grades 1-2); 2% (grade 3) Blurred vision: 10% (grades 1-2); 1% (grade 3) Retinal hemorrhage: 1% (grade 3) Bacterial conjunctivitis: 1% (grade 3)	HER2-positive ORR: *n* = 16/48 (33%) HER2-low hormone receptor-positive ORR: *n* = 9/32 (28%) HER2-low hormone receptor-negative ORR: *n* = 6/15 (40%) PR: *n* = 31/95 (33%) Confirmed PR: *n* = 23/31 (74%)
HER2-positive breast cancer (*n* = 291)^50^	1.2 mg/kg Q3W	Conjunctivitis: 38.2% Keratitis: 38.2%	NR NR
*Tusamitamab ravtansine* CEACAM5-targeting mAb conjugated to the microtubule inhibitor DM4 via a cleavable linker			
Locally advanced or metastatic solid tumors (*n* = 31)^51^	5-150 mg/m^2^ Q2W	At least one treatment-related corneal AE: 29.0% (any grade); 19.4% (grade ≥3) Keratopathy: 25.8% (all grades); 19.4% (grade ≥3) Dry eye: 12.9% (any grade) Blurred vision: 12.9% (any grade) Keratitis: 3.2% (grade 2) Punctate keratitis: 3.2% (grade ≥3)	ORR All patients: 9.7% Patients receiving 100 mg/m^2^: 33.3% Patients receiving 120 mg/m^2^: 11.1% Confirmed PR: *n* = 3/31 (9.7%)
Nonsquamous CEACAM5-positive NSCLC (*n* = 92)^52^	100 mg/m^2^ Q2W	Keratitis/keratopathy: 38% (any grade); 10.9% (grade ≥3)	ORR High CEACAM5 expression: *n* = 13/64 (20.3%) Moderate CEACAM5 expression: *n* = 2/28 (7.1%)
Nonsquamous CEACAM5-positive NSCLC (long-term treatment [≥ 12 months]) (*n* = 11)^53^	100 mg/m^2^ Q2W	Keratitis/keratopathy: 72.7% (any grade); 36.4% (grade ≥3)	Best overall response Confirmed PR: *n* = 7/11 (64%) SD: *n* = 4/11 (36%)
Advanced solid tumors (pooled analysis) (*n* = 186)^54^	100 mg/m^2^ Q2W	Ocular AEs: 30.1% (any grade); *n* = 16 (grade 3) Keratitis: 22% (any grade); 6.5% (grade 3) Keratopathy: 11.8% (any grade); 2.7% (grade 3)	NR

^a^Indicates overall response rate.

Abbreviations: ADC, antibody-drug conjugate; AE, adverse event; ALL, acute lymphoblastic leukemia; CEACAM5, carcinoembryonic antigen-related cell adhesion molecule-5; CI, confidence interval; CR, complete response; DLBCL, diffuse large B-cell lymphoma; HER2, human epidermal growth factor receptor-2; IgG1, immunoglobulin G1; mAb, monoclonal antibody; no., number; NR, not reported; NSCLC, non-small cell lung cancer; ORR, objective response rate; QW, every week; Q2W, every 2 weeks; Q3W, every 3 weeks; Q4W, every 4 weeks; PR, partial response; SD, stable disease; TNBC, triple-negative breast cancer; TRAE, treatment-related adverse event; TROP2, trophoblast cell-surface antigen 2.

### DM4

Mirvetuximab soravtansine is a folate receptor alpha (FRα)-targeting immunoglobulin G1 (IgG1) subtype 2 mAb with a DM4 payload approved in the United States (US)—but not yet in Europe—for the treatment of FRα-positive, platinum-resistant ovarian epithelial, fallopian tube, or primary peritoneal cancer in patients who have received 1-3 prior systemic regimens.^55^ It includes a boxed warning regarding the risk for ocular AEs, including visual impairment, keratopathy, dry eye, photophobia, eye pain, and uveitis.^55^

Ocular AEs were among the most common AEs reported in clinical studies of mirvetuximab soravtansine,^24-26,56^ including a phase I expansion cohort trial of 46 patients with platinum-resistant ovarian, fallopian tube, or primary peritoneal cancer^56^; the open-label phase 3 FORWARD I trial of 366 patients with platinum-resistant ovarian cancer^24^; the single-arm phase III SORAYA trial in 106 patients with FRα-positive, platinum-resistant ovarian cancer^25^; and most recently, preliminary results from the phase III MIRASOL study in 453 patients with platinum-resistant ovarian cancer.^26^ Across the phase I expansion cohort trial, FORWARD I, and SORAYA, ocular AEs were predominantly grades 1-2 and most frequently included blurred vision (41%-42%) followed by keratopathy (26%-33%) and dry eye (13%-26%).^24,25,56^ The incidence of grade ≥3 blurred vision, keratopathy, and dry eye had a range of 1%-9% in the FORWARD I and SORAYA trials.^24,25^ Across the 3 trials, the dose of mirvetuximab soravtansine was delayed or reduced in 11%-20% of patients,^24,25,56^ and across the phase I and SORAYA trials treatment was discontinued in 4 patients due to ocular AEs, such as grade 1 eye pain and corneal cysts and grade 2 blurred vision (Table 2).^25,56^ In addition, 14% of patients who received mirvetuximab soravtansine in MIRASOL had grade ≥3 ocular AEs. Objective response rates (ORRs) ranged from 22% to 42.3% across the 4 trials.^24-26,56^

Several ADCs with a DM4 payload are in recent clinical development, some of which include anetumab ravtansine, praluzatamab ravtansine, and tusamitamab ravtansine (Table 3). Anetumab ravtansine, a mesothelin-targeting IgG1 mAb,^57^ has been investigated in several cancer types, but its development was paused due to failure to meet the primary endpoint in a pivotal mesothelioma trial.^37-39^ Ocular AEs were reported in 3 trials of anetumab ravtansine, including a phase II trial in 163 patients with mesothelin-positive malignant pleural mesothelioma,^37^ a phase Ib trial in 65 patients with platinum-resistant ovarian cancer,^38^ and a preliminary report from a phase Ib trial in 33 patients with mesothelin-positive advanced pancreatic adenocarcinoma.^39^ Across the 3 trials, grades 1-2 ocular AEs included corneal disorder (29%-37%), dry eye (13%-17%), blurred vision (11%-15%), xerophthalmia (3%), and keratitis (3%-6%),^37-39^ with 48% of patients reporting at least one corneal epitheliopathy event (all grades 1-2).^38^ Grade 3 ocular AEs were observed in 2 of the trials, including corneal disorder (2%) and blurred vision (3%).^37,38^ Treatment discontinuation due to ocular AEs was rare, occurring in one patient in the phase II trial.^37^ The ORRs ranged from 0% to 27.7% across the 3 trials.^37-39^

Praluzatamab ravtansine, a probody (inert antibody until activated) drug conjugate with a CD166-targeting mAb, is in development for advanced solid tumors and was assessed in a phase I/2 trial (*N* = 99) in which 43% of patients who received praluzatamab ravtansine reported treatment-related ocular AEs, including keratitis, blurred vision, dry eye, keratopathy, photophobia, punctate keratitis (Supplementary Table S1), and eye pain, with 11% of patients having grade ≥3 ocular AEs (Table 3).^47^ Ocular AEs resulted in treatment discontinuation (*n* = 7), dose interruption (*n* = 18), and dose reduction (*n* = 2).

Tusamitamab ravtansine, composed of a carcinoembryonic antigen-related cell adhesion molecule-5 (CEACAM5)-directed mAb conjugated to the microtubule inhibitor DM4 via a cleavable linker, was investigated for the treatment of solid tumors (eg, CEACAM5-positive NSCLC and colorectal, stomach, pancreas, breast, esophageal, and gastroesophageal junction cancer)^51,53^ in an open-label, dose-escalation, dose-expansion trial. In the dose-escalation, phase I portion of the trial involving 31 patients with locally advanced or metastatic solid tumors, reversible grade 3 microcystic keratopathy was the dose-limiting toxicity, developing in 28 patients after cycle 2.^51^ Overall, 9 (29%) patients experienced at least one treatment-related ocular AE, including 6 patients with grade ≥3 keratopathy (with no grade 4 keratopathy). Dose reduction due to ocular AEs was required in 7 patients, and treatment discontinuation occurred in 1 patient due to grade 3 keratopathy.^51^

In the dose-expansion, phase Ib portion of the same trial involving 92 patients with NSCLC and high or moderate CEACAM5 expression receiving tusamitamab ravtansine 100 mg/m^2^ (maximum tolerated dose), keratopathy/keratitis was the most common AE (38% of patients, grade ≥3 in 10.9%).^52^ Dose modification due to ocular AEs was required in 27.2% of patients. Overall, 11 patients had received ≥12 months of tusamitamab ravtansine, and keratitis/keratopathy remained the most common AE in these patients (72.7%, grade ≥3 in 36.4%).^53^ Of the 11 patients, dose modification was required in 7 patients, but no ocular AE led to treatment discontinuation in this long-term treatment patient subgroup.^53^ A recent pooled analysis of ocular safety results from the same study included 186 patients with advanced solid tumors and reported corneal AEs in 56 (30.1%) patients, with keratitis (22%) and keratopathy (11.8%) being the most common.^54^ Most of these AEs were grades 1-2 and occurred within the first 4 cycles. Grade 3 corneal AEs were reported in 16 patients, including grade 3 keratitis (6.5%) and keratopathy (2.7%). Corneal AEs led to treatment delay in 15.6% of patients, with 7% reporting dose reduction. Most AEs (71.4%) resolved at the time of data cut, with a median time to recovery of 20.5 days. Importantly, no patients had a grade 4 (perforation or BCVA worse than 20/200 in the affected eye) event, and no patients discontinued treatment due to ocular AEs.^54^ ORRs with tusamitamab ravtansine 100 mg/m^2^ were 33.3% in the dose-escalation phase in patients with advanced solid tumors and 20.3% in the dose-expansion phase (patients with NSCLC and high CEACAM5 expression) (Table 3).^51,52^ CEACAM5 has not been detected on corneal tissue; thus, ocular AEs reported with tusamitamab ravtansine are likely due to off-target toxicity.

### MMAF

Like ADCs with DM4 payloads, the incidence of associated ocular AEs may be higher with MMAF than with other payload types.^3,12^

Belantamab mafodotin, a B-cell maturation antigen (BCMA)-targeting IgG1 mAb with an MMAF payload, was initially approved in the US and Europe as monotherapy for the treatment of relapsed/refractory multiple myeloma (RRMM) in patients who have received at least 4 prior therapies^58,59^ but was subsequently withdrawn from the US market due to failure as monotherapy in the DREAMM-3 trial.^60^ The US prescribing information for belantamab mafodotin included a boxed warning regarding the risk for ocular AEs, including changes in the corneal epithelium resulting in vision changes (eg, severe vision loss due to corneal ulceration) and ocular symptoms (eg, blurred vision and dry eye).^59^

Clinical trials reporting ocular AEs with belantamab mafodotin included the dose-expansion phase I DREAMM-1 trial, in which 35 patients received belantamab mafodotin 3.4 mg/kg every 3 weeks,^16^ and the pivotal phase II DREAMM-2 study, in which patients received belantamab mafodotin 2.5 mg/kg (*n* = 95) or 3.4 mg/kg (*n* = 99).^17^ Both trials reported ocular AEs,^16-18^ including 63% of patients in the dose-expansion phase of the DREAMM-1 trial.^16^ Common ocular AEs across the 2 studies included blurred vision (22%-46%), dry eye (14%-34%), photophobia (23%), increased lacrimation (11%), keratitis (9%), eye pain (6%), and keratopathy or changes to the corneal epithelium (6%-75%).^16,17^ Furthermore, slit-lamp examination revealed corneal abnormalities (eg, superficial punctate keratitis, stromal edema [Supplementary Table S1]) in 89% of patients in DREAMM-1 and microcyst-like epithelial changes in 72% (grade ≥3 46%) and impairment in BCVA in 54% of patients in DREAMM-2 with belantamab mafodotin 2.5 mg/kg.^16,18^ Grade 3 ocular AEs, including abnormal visual acuity test, keratitis, eye pain, eye disorder, and retinal detachment, occurred in 3%-6% of patients in the DREAMM-1 study, and up to 27% of patients in DREAMM-2 reported grade ≥3 keratopathy (or changes to the corneal epithelium), of which 28% had resolved to Grade ≤1.^16,17^ Blurred vision or keratopathy were the most common AEs leading to dose delay (34%-48%), dose reduction (23%-31%), or permanent treatment discontinuation (1%-3%) across the 2 studies. ORRs ranged from 31% to 60% in the 2 trials (Table 2).

Depatuxizumab mafodotin (also known as ABT-414, depatux-m) is an epidermal growth factor receptor (EGFR)-targeting IgG1 mAb with an MMAF payload, albeit its clinical development was curtailed due to a lack of survival benefit seen in a randomized phase III trial in patients with EGFR-amplified glioblastoma multiforme.^61^ Despite incorporating prophylactic ocular corticosteroids and other supportive care measures (eg, lubricating eye drops, therapeutic bandage contact lenses, punctal plugs), grades 3 and 4 corneal epitheliopathy events (keratopathy, blurred vision, photophobia, dry eye, eye pain, keratitis, and punctate keratitis) were reported in 55% and 5%, respectively, of patients randomized to depatux-m (vs 0.6% and 0% in the placebo group), and dose modification and treatment discontinuation due to these events (all grades) were required in 44% and 12% of patients, respectively.^61^

### DM1

ADCs with a DM1 payload are generally associated with fewer ocular AEs than ADCs with DM4 and MMAF payloads.^3,12^ Trastuzumab emtansine (T-DM1) is a HER2-targeting mAb with a DM1 payload and is approved for multiple indications in the US and Europe for the treatment of HER2-positive metastatic breast cancer.^34,62^ T-DM1 does not include a boxed warning regarding the risk of ocular AEs. In the T-DM1 group of the pivotal phase III EMILIA and KATHERINE clinical trials, the incidence of ocular AEs was <10% and included blurred vision (4.5% and 3.9%, respectively), conjunctivitis or dry eye (3.9% and 4.5%, respectively), and increased lacrimation (3.3% and 6%, respectively; Table 2).^34^ The ORR was 43.6% in the EMILIA trial and was not reported in the KATHERINE trial.^35,36^ Ocular AEs reported with T-DM1 may be due to on-target toxicity, as HER2 expression has been reported in normal corneal tissue.^13^

### MMAE

Approved ADCs with an MMAE payload include enfortumab vedotin and tisotumab vedotin. Enfortumab vedotin, with a nectin-4-targeting IgG1 mAb, is approved in the US and Europe as monotherapy for the treatment of locally advanced or metastatic UC in patients who have received prior platinum-based chemotherapy and programmed cell death protein 1 (PD-1) or programmed cell death ligand 1 (PD-L1) inhibitor therapy.^63,64^ In clinical trials of enfortumab vedotin, including the phase II EV-201 study (*n* = 125) and the phase III EV-301 trial (*n* = 296),^22,23^ the vast majority of ocular AEs were grades 1-2, with the most common being dry eye (15%-19%), followed by increased lacrimation (14%), blurred vision (4%-12%), and corneal disorders (1%).^22,23^ Overall, grade 3 ocular AEs were rare (<1% of patients reported grade 3 ocular AEs in the EV-301 trial; Table 2).^23^ The ORRs were 44% and 40.6% in the EV-201 and EV-301 trials, respectively.^22,23^

Tisotumab vedotin, with a tissue factor-targeting IgG1, is approved in the US for the treatment of recurrent or metastatic cervical cancer with disease progression on or after chemotherapy.^65^ The US prescribing information for tisotumab vedotin includes a boxed warning regarding the risk for ocular AEs (eg, severe vision loss and corneal ulceration).^65^ Clinical studies of tisotumab vedotin in patients with recurrent or metastatic cervical cancer included the phase I/2 innovaTV 201 trial (*N* = 55),^27^ the phase I/2 innovaTV 206 trial (*N* = 17),^28^ and the phase II innovaTV 204/GOG-3023/ENGOT-cx6 trial (*N* = 101).^29^ Grades 1-2 ocular AEs were reported in 35%-65% of patients across the 3 trials, with the most common ocular AEs being conjunctivitis (18%-42%), dry eye (23%-24%), and keratitis, including ulcerative keratitis (5%-11%) (Supplementary Table S1).^27-29^ Grade ≥3 ocular AEs were rare and included conjunctivitis (1%) and ulcerative keratitis (2%) in the innovaTV 201 and innovaTV 204/GOG-3023/ENGOT-cx6 studies, respectively.^27,29^ Treatment discontinuation due to ocular AEs (mostly due to conjunctivitis [4%]) was noted only in the innovaTV 201 trial.^27^ Tisotumab vedotin yielded robust ORRs, ranging from 24% to 29.4% across the 3 trials (Table 2).^27-29^

Some of the investigational ADCs with an MMAE payload include DLYE5953A, DMUC4064A, and lifastuzumab vedotin (also known as LIFA), all of which were once evaluated for treatment of solid tumors, but whose development was terminated due to lack of efficacy or the competitive treatment landscape.^66-69^

Ocular AEs were not prominent with DLYE5953A treatment in a phase I study (*N* = 68) and were all grades 1-2.^66^ However, in the dose-escalation study of DMUC4064A involving patients with relapsed ovarian cancer after prior therapy, treatment-emergent ocular AEs appeared to be dose-related (ranging from 1 mg/kg to 5.6 mg/kg) and included blurred vision (62% all grades; 4% with grade ≥3), dry eye (all grades 27%; no grade ≥3), and keratitis (27% all grades; 12% with grade ≥3) at the recommended phase II dose of 5.2 mg/kg (*n* = 26); the ORR was 25% across all doses.^67^

A dose-escalation trial (*N* = 41) of the combination of LIFA with carboplatin (with or without bevacizumab) reported blurred vision (all grades 20%; no grade ≥3) and cataract (all grades 12%; grade ≥3 10%)^69^; the ORR was 59% for combination therapy.

### Novel auristatins

ZD02044 is a novel N-acyl sulfonamide auristatin that is currently used as a payload in several ADCs in clinical development, such as XB002, which was associated with treatment-emergent ocular AEs in 42% of patients, including dry eye (16%) and noninfective conjunctivitis (26%) in the ongoing dose-escalation JEWEL-101 study. Ocular events appear to be dose-related and reversible.^70-72^

### Topoisomerase I inhibitors

Trastuzumab deruxtecan (T-DXd) is a HER2-targeting IgG1 mAb with a topoisomerase I inhibitor payload approved in the US and Europe for treating multiple solid tumors, including breast cancer, gastric or gastroesophageal junction adenocarcinoma, and NSCLC.^31,73,74^

Ocular AEs were not frequently reported with T-DXd in clinical studies of patients with HER2-positive breast cancer, including in DESTINY-Breast01 (*N* = 184), DESTINY-Breast03 (*N* = 257), and DESTINY-Breast04 (*n* = 371) (Table 2).^30,32,33^ Common ocular AEs across the 3 studies included dry eye (11.4%) and blurred vision (3.5%-4.9%), with one (0.5%) patient reporting grade 4 dry eye in DESTINY-Breast01.^30–33^ ORRs were favorable across the 3 studies, ranging from 52.3% to 79.7%.

Datopotamab deruxtecan (Dato-DXd), a trophoblast cell-surface antigen 2 (TROP2)-directed IgG1 mAb with a topoisomerase 1 inhibitor payload, is in clinical development for the treatment of NSCLC and triple-negative breast cancer (TNBC).^44,75,76^ Preliminary data from the TROPION-Lung01 and TROPION-Lung02 studies involving patients with NSCLC reported ocular surface toxicity in up to 24% of patients receiving Dato-DXd plus pembrolizumab and chemotherapy in the TROPION-Lung02 study,^45^ and ocular AEs in 19% of patients (all grades; 2% with grade ≥3) receiving Dato-DXd in the TROPION-Lung01 study,^46^ with ORRs for any line of therapy ranging from 26% to 49% across the 2 studies.^45,46^ TROP2 expression has been detected in healthy corneal tissue,^77^ which has important implications for the mechanism of ocular AEs following treatment with Dato-DXd.

### AS269 and duocarmycin

Currently, no ADCs with AS269 or duocarmycin payloads are approved for clinical use in the US; however, ARX788 and trastuzumab duocarmazine—ADCs with AS269 and duocarmycin payloads, respectively—are in clinical development for locally advanced or metastatic solid tumors.^40,49,50^ In a phase I study (*N* = 69) of patients with HER2-positive metastatic breast cancer, ocular AEs with ARX788 included corneal epitheliopathy (46%; 4% with grade ≥3), blurred vision (22%; 3% with grade ≥3), and xerophthalmia (22%; all grades 1-2) (Table 3), which were all reversible. Treatment discontinuation due to ocular AEs was rare (2 patients). ARX788 showed promising antitumor activity, yielding an ORR of 65.5%.^40^

In the dose-expansion cohort of a phase I study (*N* = 146) of trastuzumab duocarmazine in patients with locally advanced or metastatic solid tumors, including HER2-positive breast cancer, 71% had at least one ocular AE, the most common being conjunctivitis and dry eye (31% each).^49^ Grade ≥3 ocular AEs, including conjunctivitis, keratitis, dry eye, blurred vision, retinal hemorrhage, and bacterial conjunctivitis, occurred in 7.5% of patients.^49^ The ORRs in the breast cancer cohorts (*n* = 95) were 28%-40%, depending on the extent of HER2 expression (Table 3).^49^ As previously mentioned, HER2 has been detected in corneal tissue; thus, ocular AEs reported with trastuzumab duocarmazine may potentially be attributed to on-target toxicity.^13^

## Recommendations for the prevention and management of ADC-associated ocular AEs

Currently, there are no consensus guidelines for the prevention and management of ADC-associated ocular AEs; however, recommendations are available for several ADCs that are known to cause ocular AEs, either in the product labeling information for approved ADCs^55,58,59,63-65^ or in other study reports or review articles.^51,78,79^ These recommendations are summarized in Table 4.

**Table 4.** Strategies for prevention and management of ocular AEs with specific ADCs in clinical use.^55,58,59,63-65^

Recommendations	Belantamab mafodotin	Enfortumab vedotin	Mirvetuximab soravtansine	Tisotumab vedotin
Prophylaxis				
Preservative-free artificial tears or lubricating eye drops	✔	✔	✔	✔
Ophthalmic topical corticosteroids before and during treatment			✔	✔
Vasoconstrictor eye drops immediately before each infusion				✔
Use cooling eye pads during infusion				✔
Avoid contact lenses unless directed by an ophthalmologist	✔		✔	✔
Monitoring				
Ophthalmic examination (visual acuity and slit lamp) prior to initiation	✔		✔	✔
Regular ophthalmic examinations (visual acuity and slit lamp)	✔	✔	✔	✔
Advise patients to report any visual changes		✔	✔	✔
Management				
Ophthalmic examination (visual acuity and slit lamp) promptly for worsening symptoms	✔	✔	✔	✔
For moderate or severe ocular AEs, withhold treatment until improvement, then restart at same or reduced dose, or consider permanent discontinuation for worsening symptoms that are unresponsive	✔	✔	✔	✔
Consider ophthalmic topical corticosteroids if indicated after ophthalmic examination		✔		

Abbreviations: ADC, antibody-drug conjugate; AE, adverse event.

### Prophylaxis

Several measures are proposed to mitigate ADC-associated ocular AEs^55,58,59,63-65^ and have been adopted in clinical studies (Table 2). Patients should avoid wearing contact lenses unless directed by their ophthalmologist. Prophylactic ophthalmic topical corticosteroids,^55,65^ ophthalmic topical vasoconstrictor drops,^65^ and the use of cooling eye pads^65^ have been recommended with certain ADCs (eg, mirvetuximab soravtansine, tisotumab vedotin). Preservative-free lubricating eye drops or artificial tears as prophylaxis for dry eye are recommended during treatment with belantamab mafodotin, enfortumab vedotin, mirvetuximab soravtansine, or tisotumab vedotin.^55,58,59,63-65^

Prophylactic measures have shown mixed effectiveness in reducing ocular AEs in clinical studies. The use of ophthalmic topical corticosteroids during treatment with tisotumab vedotin reduced the incidence of conjunctivitis in the innovaTV 201 study,^80^ forming the basis for recommending prophylactic corticosteroid eye drops prior to and for 72 h after tisotumab vedotin infusion.^78^ However, prophylactic corticosteroids did not reduce the incidence of keratopathy in patients with FRα-positive ovarian cancer who received ophthalmic topical corticosteroid drops during mirvetuximab soravtansine treatment,^81^ and in patients with RRMM receiving belantamab mafodotin in the DREAMM-2 study.^17^ Furthermore, a Phase I study of tusamitamab ravtansine in patients with advanced solid tumors and a pooled analysis of the same study demonstrated that keratopathy prophylaxis, including lubricating eye drops, vasoconstrictor eye drops, ocular corticosteroid gel, and cooling pads, was not beneficial in preventing the development of keratopathy.^51,54^ Oncologists should consider implementing these prophylactic measures in patients with a known risk of ocular AEs (ie, history of keratopathy).

### Monitoring

Proactive monitoring for ocular AEs by oncologists is essential.^78^ Some ADCs require ophthalmic examination (including visual acuity and slit-lamp examination) by an ophthalmologist prior to treatment initiation, before each infusion, and upon appearance or worsening of ocular symptoms (Table 4).^55,58,59,65,79^ Prompt ophthalmologist referral may be required for the diagnosis, grading, and management of ocular AEs, particularly those that do not resolve or that worsen.^63,64,78^ For example, the DREAMM-2 study demonstrated the utility of slit-lamp examination and in vivo confocal microscopy to characterize microcyst-like epithelial changes associated with belantamab mafodotin treatment in patients with RRMM.^18^ Following referral, oncologists should continue to monitor patients for improvement or worsening of ocular symptoms, including decreased vision, and remain in communication with the ophthalmologist regarding the need for dose modifications or treatment discontinuation.^78^ Because monitoring recommendations vary across approved ADCs, oncologists should refer to the relevant prescribing information.

### Management

#### Dose modifications

Ocular AEs can often be managed with dose modification (interruption or reduction) or treatment discontinuation of the ADC, depending on the type, persistence, and severity of the ocular AE. Ocular AEs, including abnormal corneal findings on ophthalmic examination, changes in visual acuity, or conjunctivitis, should be managed by treatment interruption in patients with grade ≥2 AEs until improvement to grade ≤1, with treatment resumed at a reduced dose.^55,58,59,65^ Dose delays and dose reductions were reported in several studies of ADCs, with most patients remaining on treatment and recovering from the ocular AEs following dose modification and close monitoring.^18,23-25,27,79^ Permanent treatment discontinuation should be considered in patients with grade 4 ocular AEs, including corneal epithelial defect, severe superficial keratopathy, ulcerative keratitis, or corneal perforation. Although rare, treatment discontinuation due to ocular AEs has been reported in ADC trials.^25,27^ As mentioned previously, dose modification recommendations may vary based on the individual ADC, so it is important to refer to the prescribing information of the approved ADC.^18,23,24,27,79^

#### Available treatments

Ophthalmologists may utilize ophthalmic-specific treatments for different ocular AEs, including topical preservative-free lubricating eye drops for dry eye or delayed corneal epithelial healing; ophthalmic topical corticosteroids for non-infectious conjunctivitis or keratitis; and ophthalmic topical antibiotics for corneal epithelial defects or ulcers.^82^ Caution must be exercised with the long-term use of topical corticosteroids given the risks of infection and eye pressure elevation.^83^ Based on our anecdotal experience, patients with dry eye and concurrent whorl-like staining and/or superior limbic keratoconjunctivitis may be treated with preservative-free artificial tears 4-6 times daily, oral doxycycline 20 mg twice daily, vitamin A ointment applied nightly at bedtime, and limited application of topical steroid (eg, around the time of infusions). If a topical steroid is to be considered on an ongoing basis, patients should be informed of potential complications and the need for regular follow up with an eye care provider.^84^ Other options to consider for corneal disorders include therapeutic bandage contact lenses or scleral contact lenses.^78^ Further studies are needed to characterize the underlying pathogenesis of ocular AEs for various agents and to determine whether available treatment options are likely to be of benefit.

### The role of the oncologist

It is important that the oncology care team is aware of the potential risk for ocular AEs with some ADCs.^79^ Oncologists should educate themselves, the broader oncology care team, and their patients regarding the potential for ocular AEs and how to appropriately monitor for and manage such events.^78,79^ This understanding will help oncologists to optimize dosing, which may lead to fewer treatment interruptions and an increased likelihood of improved clinical outcomes.^78^

The oncology care team should emphasize to their patients the potential risks and the importance of reporting ocular symptoms, particularly blurred vision, dry eye symptoms, and loss of visual acuity, and routinely ask whether any of these ocular symptoms impact their quality of life (eg, blurred vision that limits reading or causes driving difficulties).^79^ The oncologist should also review the patient’s eye examination findings prior to dosing and determine the most appropriate therapeutic strategy based on the most severe grade of findings. Therefore, close communication with the managing ophthalmologist is crucial for ensuring treatment continuation, with incorporation of dose reduction or delay if required to manage ocular AEs.^79^

## Conclusions

Several ADCs in clinical use or in development are associated with ocular AEs, although the exact mechanisms of these events are not fully understood. Attributed in part to the off-target effects of ADCs, ocular AEs are more commonly reported with ADCs that utilize DM4 or MMAF vs MMAE or DM1 payloads.^3,12^ Nevertheless, the ability to manage the risk of ocular AEs with ADCs should be weighed against benefits, as some ADCs, including mirvetuximab soravtansine and tusamitamab ravtansine, have yielded favorable ORRs.

In most cases, ocular AEs are consistent with corneal changes (eg, causing symptoms of blurred vision due to keratopathy) and appear irrespective of the cellular targets of the individual ADC.^12^ Most ocular AEs are reversible or manageable with dose interruption or modification and do not require treatment discontinuation. This is important, as treatment discontinuation, especially permanent discontinuation, can negatively affect treatment outcomes.^85,86^

Effective management of ocular AEs to minimize treatment discontinuation requires the involvement of a multidisciplinary care team and close collaboration between oncologists and ophthalmologists. However, limited data are available concerning the effectiveness of the available prevention and management strategies for ocular AEs, and future studies are warranted. These ocular AEs may require management strategies that are new to oncology care teams, highlighting the need for further education of the oncologist and the oncology care team.

## Supplementary Material

Supplementary material is available at *The Oncologist* online.

oyae177_supplSupplementary_Table_S1

## Data Availability

The data underlying this article are available in the article and in its online supplementary material.
