# Supplementary material for: Ocular adverse events associated with antibody-drug conjugates for cancer: evidence and management strategies
Source: Oncologist. 2024 Jul 24;29(11):e1435–51. doi: 10.1093/oncolo/oyae177 (PMC11546764; doi:10.1093/oncolo/oyae177)
Supplement: oyae177_supplSupplementary_Table_S1 [file oyae177_supplsupplementary_table_s1.docx]

# Supplementary Materials

## Supplementary Table 1.

**.** Definitions of Additional Eye Conditions

| **Superficial punctate keratitis** | Death of small groups of cells on the surface of the cornea; characterized by red, watery, and sensitive eyes (to light), with possible vision impairment.^1^ |
| --- | --- |
| **Stromal edema** | Manifests as thickening of the corneal stroma, resulting in a mild-to-moderate reduction in visual acuity.^2^ |
| **Retinal exudates** | Lipid residues that leak from damaged capillaries. |
| **Ulcerative keratitis** | An open sore at the outer layer of the cornea.^3^ |
| **Allergic conjunctivitis** | An inflammatory response of the conjunctiva to an allergen.^4^ |
| **Scleritis** | Severe ocular inflammatory condition affecting the outer cover of the eyes.^5^ |
| **Hordeolum (stye)** | Red bump on the edge of the eyelid, commonly caused by a bacterial infection.^6^ |
| **Punctate epitheliopathy** | Dot-like staining of the corneal epithelium; may manifest as sharp pain, tearing, redness, and decreased vision.^7^ |

**References**

1. Roat MI. Superficial punctate keratitis. <https://www.merckmanuals.com/professional/eye-disorders/corneal-disorders/superficial-punctate-keratitis?query=superficial%20punctate%20keratitis>. Accessed June 9, 2023.

2. Dawson DG, Edelhauser HF. Corneal edema. In: Levin LA, Albert DM, eds. *Ocular Disease.* Elsevier; 2010.

3. Corneal ulcer. <https://www.pennmedicine.org/for-patients-and-visitors/patient-information/conditions-treated-a-to-z/corneal-ulcer>. Published 2023. Accessed June 9, 2023.

4. Syed ZA. Allergic conjunctivitis. <https://www.merckmanuals.com/professional/eye-disorders/conjunctival-and-scleral-disorders/allergic-conjunctivitis>. Published 2023. Accessed June 9, 2023.

5. Lagina A, Ramphul K. *Scleritis.* Treasure Island, FL: StatPearls Publishing; 2022.

6. Hordeolum (stye). <https://www.hopkinsmedicine.org/health/conditions-and-diseases/hordeolum-stye>. Updated 2023. Accessed June 9, 2023.

7. Qu JH, Li L, Tian L, Zhang XY, Thomas R, Sun XG. Epithelial changes with corneal punctate epitheliopathy in type 2 diabetes mellitus and their correlation with time to healing. *BMC Ophthalmol.* 2018;18(1):1.
